# Pediatric COVID-19 patient with exacerbated generalized pustular psoriasis

**DOI:** 10.1590/0037-8682-0318-2021

**Published:** 2021-09-24

**Authors:** Erdal Pala, Mehmet Melikoğlu, Merve Hatun Erkayman

**Affiliations:** 1 Atatürk University, Faculty of Medicine, Department of skin and venereal diseases Erzurum, Turkey.

A 12-year-old male patient was diagnosed with plaque psoriasis when he was 5 years old and has been in remission for about 7 years. The patient, who is in remission, has new lesions that have appeared in the last week. On dermatological examination, we observed multiple pustular lesions on an erythematous background on the upper extremities and the trunk (Figure A). Biopsy was performed following the preliminary diagnosis of generalized pustular psoriasis (GPP).

The histopathological findings were consistent with GPP (Figure B and Figure C).

**FIGURE A:**
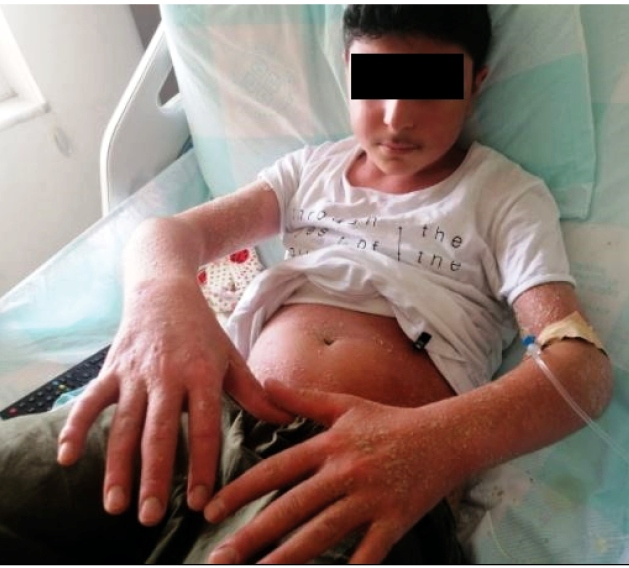
Numerous millimetric pustular lesions with erythematous bases at the upper extremities and trunk.

**FIGURE B:**
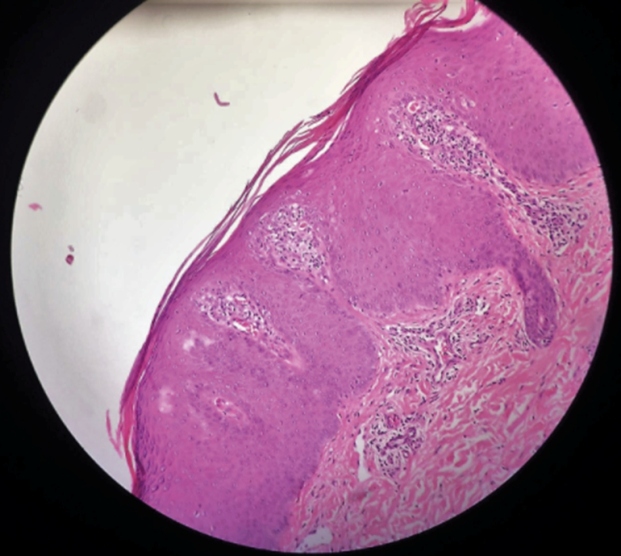
Acanthosis, thinning of the suprapapillary plate, parakeratosis, round veins on the papilla tops, papillomatosis (H&Ex10).

**FIGURE C:**
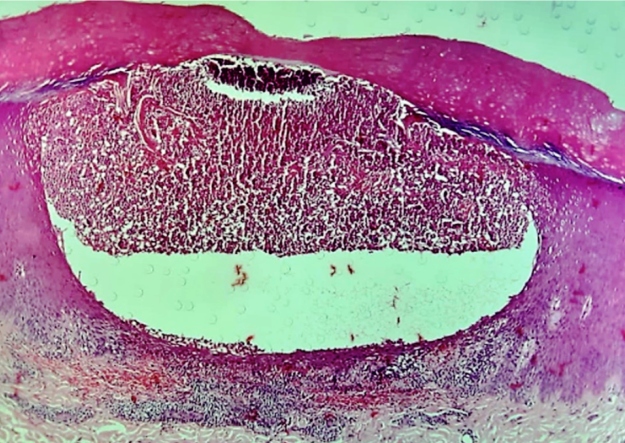
Subcorneal pustule formation rich in neutrophils (H&Ex20).

Polymerase chain reaction (PCR) test was performed to screen for COVID-19 infection in the patient owing to the complaints of high fever and cough during follow-up. Since the test result was positive, the patient was transferred to the pediatric infectious diseases polyclinic.

COVID-19 infection is a pandemic that has turned into a global problem. The number of dermatological diseases that are known to coexist with this infection has been increasing with the accumulation of cases.

Coronaviruses (CoVs) are positively polarized, single-stranded, enveloped ribonucleic acid (RNA) viruses of the Coronaviridae family. Pustular psoriasis is a rare subtype of psoriasis. Although its etiopathogenesis is not clear, it is assumed that it appears in genetically vulnerable people through immunological and environmental factors. Recently, it is thought that psoriasis could be a new, genetic, autoimmune disease associated with interleukin (IL)-36 receptor mutation^1^.

Pregnancy, drug use, infections, and sudden discontinuation of irritating topical substances, and corticosteroids that are used in plaque-type psoriasis have been defined as triggering factors for GPP^2^. 

Many viruses that cause upper respiratory tract infections have been held responsible for psoriasis^3^. 

We believe that the severe acute respiratory syndrome coronavirus 2 (SARS-CoV-2) might also play a role in the etiopathogenesis of pustular psoriasis through the stimulation of various inflammatory cytokines, especially IL-36.

Few cases of co-existence of pustular psoriasis and COVID-19 have been reported. This is the first report of a pediatric patient who developed pustular psoriasis due to COVID-19 infection.
